# A simple computational model for scleral stiffness assessments via air-puff deformation OCT

**DOI:** 10.3389/fbioe.2024.1426060

**Published:** 2024-07-30

**Authors:** Andres De La Hoz, Lupe Villegas, Susana Marcos, Judith S. Birkenfeld

**Affiliations:** ^1^ Instituto de Óptica “Daza de Valdés”, Consejo Superior de Investigaciones Científicas (IO-CSIC), Madrid, Spain; ^2^ Center for Visual Science, The Institute of Optics, Flaum Eye Institute, University of Rochester, Rochester, NY, United States

**Keywords:** OCT, sclera, finite element, air-puff, myopia

## Abstract

**Introduction:** The mechanical properties of the sclera are related to its structural function, and changes to these properties are believed to contribute to pathologies such as myopia. Air-puff deformation imaging is a tool that uses an imaging system coupled with an air-puff excitation source to induce and measure deformation in a tissue *in vivo*. Typically used for the study of the cornea’s mechanical properties and IOP, this tool has been proposed as a method to evaluate scleral stiffness.

**Methods:** In this work, we present a computational model of the rabbit eye to assess scleral deformation under air-puff. Parametric studies were conducted to evaluate the effects of material properties, intraocular pressure, and other parameters on the deformation response. Output from the model was also compared to experimental measurements of air-puff deformation in rabbit eyes under varying IOP.

**Results:** Central deformation response was found to be most influenced by material properties of the sclera (at site of air-puff and posterior), thickness, and IOP, whereas deformation profile was most influenced by material properties. Experimental and simulated IOP dependence were found to be similar (RMSE = 0.13 mm).

**Discussion:** Scleral APDI could be a useful tool for quick *in vivo* assessment of scleral stiffness.

## 1 Introduction

The sclera is the eye’s principal load-bearing tissue and determines the size and shape of the eye. It consists of bundled collagen fibers (Keeley et al., 1984), irregular in thickness and density, with preferential orientation and high stiffness (Coudrillier et al., 2013) embedded in an extracellular matrix composed of proteoglycans, glycosaminoglycans, water, and other molecules. The sclera’s properties are of particular interest in the study of pathologies that involve the sclera’s structural function. Myopia (near-sightedness) is one such pathology, typically caused by an excessive ocular axial elongation. Myopia has been associated with scleral thinning (Curtin and Teng, 1958), changes in collagen fibril diameter (McBrien et al., 2001) and orientation (Markov et al., 2018) and extracellular matrix composition (McBrien et al., 2000; Rada et al., 2000). Changes in mechanical behavior of myopic scleral tissue have been observed, such as differences in elastic (Phillips and McBrien, 1995) and viscoelastic (Phillips et al., 2000) properties. Cross-linking has been proposed as a method to slow down the progression of the disease (Wollensak and Spoerl, 2004) by stiffening the tissue and reducing axial growth (Dotan et al., 2014; Liu et al., 2016).

The study of the sclera’s mechanical properties can be approached with standard mechanical testing tools (such as uniaxial tensile testing). A number of more complex techniques have been proposed. Examples include inflation (sometimes combined with wide-angle X-ray scattering (Coudrillier et al., 2012; Grytz et al., 2014; Coudrillier et al., 2015), Brillouin microscopy (Shao et al., 2016), and Optical Coherence Elastography (Zvietcovich et al., 2020; Ramier et al., 2020; Villegas et al., 2024). These methods have been used to characterize isotropic and anisotropic properties of the sclera.

Air-puff deformation imaging (APDI) is a technique that involves the use of a rapid, controlled pulse of air (or “air-puff”) that induces deformation of the ocular tissue over few milliseconds and which is coupled to an imaging system that images the resulting deformation. Commercially used corneal APDI, typically coupled with a Scheimpflug camera, is mainly used to measure intraocular pressure (Corvis ST, Oculus, Wetzlar, Germany). Laboratory-based APDI systems using high-speed OCT for corneal imaging have been demonstrated for the study of the cornea’s (Dorronsoro et al., 2012; Bronte-Ciriza et al., 2021) mechanical response, and recently in the sclera (Bronte-Ciriza et al., 2021). The estimation of the material parameters is based on the quantified changes of the recorded tissue deformation images, and is often aided by the use of finite element (FE) modelling, a widely used numerical simulation method for the study of the mechanical behavior of structures under load. In this procedure, an FE model of the tissue (or the entire ocular globe) is subjected to a defined air-puff pressure load, and the resulting deformation is calculated. This approach has been used to estimate the effect of the corneal elastic modulus, intraocular pressure (IOP), and central corneal thickness (CCT) on the corneal deformation under air-puff (Kling et al., 2014; Ariza-Gracia et al., 2015). The insights obtained from such models can be used to estimate material properties based on easily-quantifiable APDI outputs (Joda et al., 2016; Chen et al., 2019; Eliasy et al., 2019). FE models can also be used to estimate material parameters by matching the output of a simulation with experimental results, a procedure known as inverse analysis. This approach has been used to estimate material properties using APDI (Bekesi et al., 2016; Bekesi et al., 2017) and other techniques such as inflation (Grytz et al., 2014; Coudrillier et al., 2015).

FE-aided approaches require making choices about the type of material model used, and thus the type of information that can be retrieved. For the sclera, the simplest approach has been the use of an isotropic material model (Asejczyk-Widlicka et al., 2007; Norman et al., 2011; Nguyen et al., 2019; Geragthy et al., 2020). Anisotropic models that treat the extracellular matrix as isotropic and the collagen fibers as having preferential direction have also been used in various inverse modeling approaches (Coudrillier et al., 2012; Grytz et al., 2014; Coudrillier et al., 2015; Jia et al., 2017; Schwaner et al., 2020), sometimes requiring additional experimental input to define this preferential direction (Coudrillier et al., 2015). An additional consideration is whether to implement spatial variation in the sclera, which can be done in various ways, e.g., modeling the sclera in discrete segments (Geragthy et al., 2020). The more complex approaches, e.g., anisotropic models, provide detailed information on the sclera, at the expense of higher computation times and limiting the application to *ex vivo* experimental work. APDI has traditionally used an isotropic model, allowing for an assessment of macro stiffness that is more easily transferable to study of live subjects and medical practice, at the expense of detail.

We have previously presented the use of APDI on the sclera (Bronte-Ciriza et al., 2021). In the current work we analyzed the differences between air-puff deformation behavior in scleral and corneal tissues, and estimated material parameters using an FE model with fixed dimensions and IOP. The extensive literature on corneal APDI indicates that the interplay between dimensions, IOP, and material affects the deformation response. Uncoupling these effects is the main challenge in assessing material properties for multiple subjects.

This current study attempts to account for the effects of expected differences across eyes, such thickness, IOP, materials, and dimensions of the ocular globe, to estimate the relative impact of these on the estimates of the scleral air-puff deformation response. These effects are studied in an FE model of the New Zealand rabbit eye subjected to a defined air-puff pressure. Two parametric studies were conducted using this FE model. In the first, a sensitivity study, a single model parameter is set as a variable and the remaining parameters are held constant. This allows the influence of each parameter on the deformation to be assessed. In the second, a random sampling study was conducted, all model parameters are set as variables and each is assigned a random value. This allows an assessment of the interplay between the input parameters and the relative strength of each in determining the scleral deformation response. Finally, the FE model was compared to experimental data of scleral air-puff deformation of *ex vivo* rabbit eyes.

## 2 Methods

### 2.1 Finite element model

#### 2.1.1 Geometry

A FE model of a New Zealand rabbit eye globe was developed in ANSYS Mechanical, v.2020 (Canonsburg, Pennsylvania). The eye was modelled on one side of the sagittal plane (assuming symmetry on the plane) and one side of the axial plane, in order to reduce computational demand. The sclera was assumed to vary in thickness across the eye, with the posterior end being the thickest part. The dimensions of the model, described in Table 1, are selected on the basis of measurements of *ex vivo* rabbit eyes in our laboratory. The exterior of the ocular globe is modelled as an ellipse with two diameters, the vertical corresponding to equatorial length (EL) and the horizontal one corresponding to axial length (AL), and the interior of the ocular globe is modelled with another ellipse using the variable of Thickness (at equator) and a fixed horizontal thickness of 0.6 mm. The cornea is modelled at an angle of 120° from the optic nerve, with a fixed thickness of 0.4 mm, and its apex extends 1 mm from the exterior ellipse such that the axial length equals the horizontal diameter of the ellipse +1.

**TABLE 1:** Direct and indirect model input parameters evaluated in the parametric study.

Model input parameters	Abbreviation	Initial	Interval
“Direct” parameters			
Material coefficient (MPa)	μ	0.083	0.06–0.10
Scleral thickness at air-puff location (mm)	THK	0.35	0.34–0.40
Intraocular pressure (mmHg)	IOP	18	10–25
“Indirect” parameters			
Posterior material coefficient (MPa)	μ_post_	0.083	0.02–0.10
Axial length (mm)	AL	18	17–19
Equatorial length (mm)	EL	19	18.4–19.6

#### 2.1.2 Material model

The material model used in this FE model is the first-order Ogden hyperelastic isotropic model. Assuming incompressibility, the stress-strain relationship under uniaxial tension for this model reduces to:

$$\sigma_{1}=\mu \left(\lambda^{\alpha }-\lambda^{-\frac{1}{2}\alpha }\right)$$ (1)

where *σ* is the stress, λ is the stretch ratio (strain+1), and α and μ are the models’ coefficients. In this model, parameter α is an exponent which influences the shape of the stress-strain curve and parameter μ influences its magnitude. The shape of the stress-strain is associated with the shape of the time profile of the air-puff deformation (displacement as a function of time). In our previous work (Bronte-Ciriza et al., 2021), the normalized time profile of various scleral regions under air-puff was found to be largely constant, suggesting a similar shape for the stress-strain curves. Based on this finding, the value of parameter α is fixed in this model, and parameter μ is treated as the variable, describing the magnitude of the stress response. Parameter α was assigned a value of 40 on the basis of approximating the shape of experimental stress-strain scleral rabbit curves and displacement time profiles.

The sclera’s material properties are known to vary spatially, which has been implemented in the model. The stiffness of the sclera is affected by physiological factors such as GAGs content (Pachenari and Hatami-Marbini, 2021) and is known to decrease from the anterior to the posterior regions (Elsheikh et al., 2010). However, fully mapping the gradient of this change is limited by technical challenges (such as sample size for uniaxial testing). In this model we have assumed the spatial variations are linear, and implemented them by setting the material coefficient of the scleral elements as a function of their position, using the following formula:

$$\mu \left(x\right)=\mu -\left(∆\mu \right)\times \frac{x}{x_{f}}$$ (2)

In this formula (Eq. 2), x = central position of the element, x_f_ = distance from equator (x = 0) to the most posterior distance of the sclera, and $∆\mu =\mu -\mu_{\text{post}}$ , where μ is the material coefficient at the equator, and $\mu_{\text{post}}$ is the material coefficient at x_f_.

#### 2.1.3 Boundary conditions, loads, and solution

The model is meshed with SOLID186 elements for the sclera and cornea and HSFLD16 for a total of 5,188 elements. Several boundary conditions and loads are required to complete the model: displacement constraints, intraocular pressure (IOP), and pressure load. Displacement constraints are as follows: symmetry on XY plane, fixed Y-displacement at XZ plane, and fixed displacements at node corresponding to most posterior point of the ocular globe.

The IOP is applied in the first loading step using HSFLD16 hydrostatic fluid elements which fill the interior of the eye. The applied pressure deforms the entire ocular structure, with the extent of the deformation depending on the magnitude of the pressure and the material properties. To account for the effect of this pressure on the initial geometry, an iterative “stress-free” procedure was introduced, adapted from the work by Elsheikh et al. (2013). The procedure begins with a predetermined geometry X_0_. IOP and scleral material properties are applied to the model, and the deformation resulting from applied IOP is extracted (u_0_). This deformation u_0_ is then subtracted from X_0_ to create a new geometry X_i_. This new geometry is used as the undeformed geometry in a new model, to which the pressure and material properties are then applied to obtain a new deformed geometry x_i_. The difference between x_i_ and X_0_ is calculated, and subtracted from stress-free form X_i_, to create X_i+1_. This process was repeated for three iterations. This ensured a consistent geometry even with varying IOP.

The air-puff is applied as a quasi-static load, in 6 load steps totaling 20 ms. The air-puff magnitude has been previously characterized and quantified (Bronte-Ciriza et al., 2021) as a spatially and temporally varying pressure load, with a maximum pressure of 15.4 kPa at 20 ms. The unloading region of the deformation event is not considered. The load is applied in the equatorial region of the sclera, centered at the uppermost point (described in Section 2.2.3).

After solving the FE model, the position of each node at every load step is exported into a.txt file and processed in MATLAB (Mathworks, Natick, MA).

#### 2.1.4 Input parameters of finite element model

The input parameters (or variables) for the model were selected on the basis of their potential relevance to the deformation response. The initial values and evaluated ranges were selected from laboratory measurements and literature data.

The input parameters can be divided into “direct” and “indirect” (see Table 1). “Direct” parameters are those acting directly on the air-puff excitation point. These are: the material coefficient, the scleral thickness at the location of air-puff, and the IOP (typically parameters evaluated as well in corneal APDI). “Indirect” model parameters are not directly acting on the air-puff excitation point, and include the axial and equatorial length of the eye, and the posterior material coefficient (see Eq. 1). Indirect parameters are expected to have less influence on the deformation response. The posterior material coefficient μ_post_ is applied as a ratio of the material coefficient ranging from 0.25 to 1, such that μ_post_ ≤ *μ*.

**FIGURE 1:**
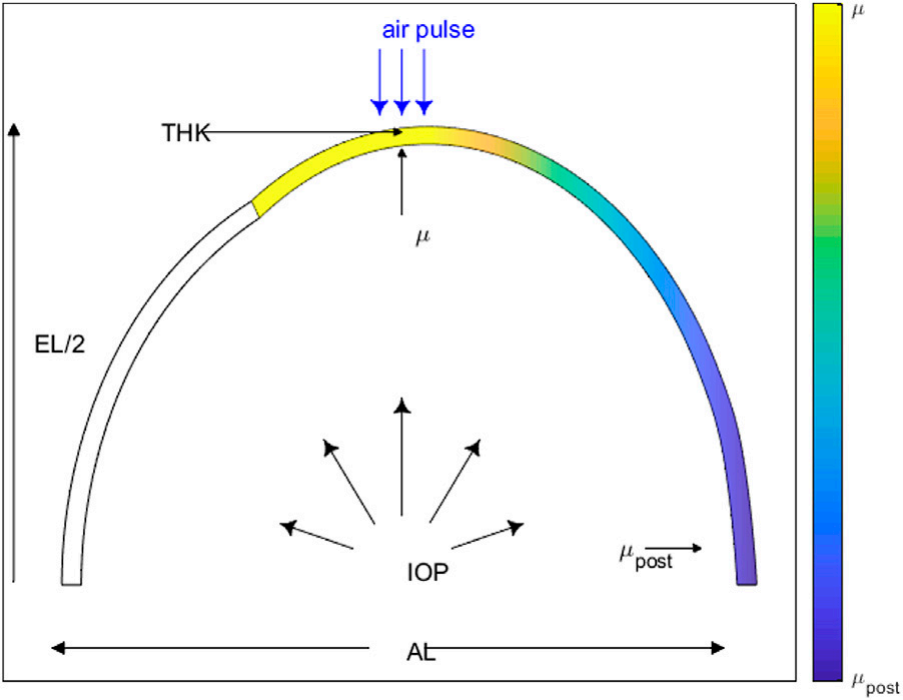
Rabbit eye model schematic displaying the FE model input parameters: material coefficient (μ), posterior material coefficient (μ_post_), intraocular pressure (IOP), thickness (THK), axial length (AL) and equatorial length (EL).

The initial value of the material coefficient was chosen so that when the model was evaluated with the initial values of the other input parameters, the Apex Displacement (AD) value was 1 mm (see Section 2.1.5 *Output parameters of Finite Element Model*). The range was selected to obtain AD values from 0.5–2.5 mm. The initial value of the posterior material coefficient was set to μ_post_ *= μ.* The range was selected so that the ratio of μ_post_/μ = 0.25–1. The initial thickness was set at 0.35 mm and evaluated in a range of 0.34–0.40 mm, on the basis of laboratory measurements and expected age-related variations (Barathi et al., 2002). Axial and equatorial length were set at 18 and 19 mm and evaluated at ranges of ±0.5 mm from initial value, on the basis of laboratory measurements, estimation from experimental data (Barathi et al., 2002), and standard deviation of measured eyes (Bozkir et al., 1997). Initial IOP was set at 15 mmHg and evaluated at ranges 10–25 mm, on the basis of IOP variation range in rabbits (Vareilles et al., 1977) and on the experimental protocols used for inflation testing. All input parameters are listed in Section 2.2.1. A schematic diagram of the rabbit eye model with the FE model input parameters is displayed in Figure 1.

#### 2.1.5 Output parameters of finite element model

After solving the FE model, the position of each node at every loading step is exported to a.txt file and processed in MATLAB (Mathworks, Natick, MA). The results are used to calculate the output parameters of the FE model.

**FIGURE 2:**
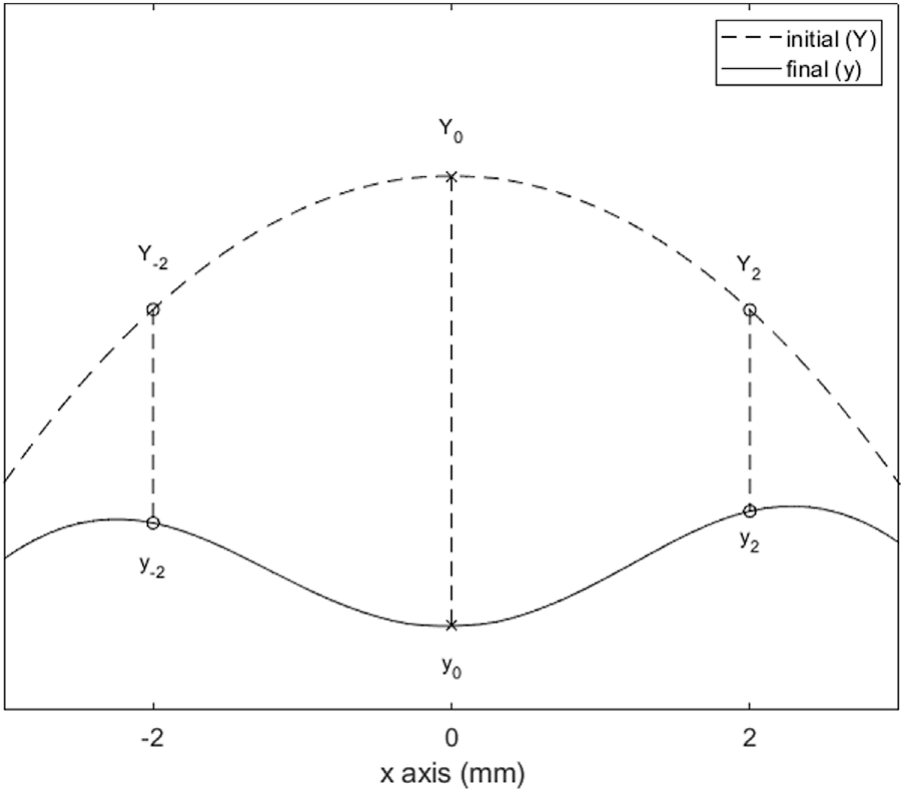
Relevant deformation points of the sclera after air-puff loading. The difference between initial (Y) and final (y) heights at x = −2, 0, and 2 mm are used to calculate the output parameters (defined in Table 2).

The parameters are defined in order to assess spatial variation of scleral deformation under air-puff. For the analysis, the sagittal cross-section is used. For convenience, we defined the most anterior point of the location that was to be scanned as the local “scleral apex”, and define its x-coordinate as 0. This is also the location where the air-puff is centered. Three displacements were estimated, as the difference between the y-coordinates at initial (Y) and final (y) step of the air-puff at the following x-coordinates: at x = 0 mm, x = −2 mm, and x = +2 mm (as shown in Figure 2). From this data, three output parameters are calculated: 1) the maximum apex displacement (AD), 2) the central peripheral ratio (CPR), and 3) the asymmetry ratio (AR). The parameter definitions are given in Table 2.

**TABLE 2:** Output parameters evaluated in the parametric study.

Model output parameters	Abbreviation	Definition
Apex displacement (mm)	AD	Y_0_-y_0_
Central peripheral displacement (mm/mm)	CPR	(Y_0_-y_0_)/(Y_2_-y_2_)
Asymmetry ratio (mm/mm)	AR	(Y_2_-y_2_)/(Y_−2_-y_−2_)

CPR and AR are considered relevant because of the non-uniform attributes of the sclera (varying thickness, decreasing material coefficient along the axial length), which will result in asymmetric deformation.

### 2.2 Parametric studies

The FE model of the rabbit eye was used for three separate analyses: 1) the evaluation of the effect and influence of individual input parameters on the deformation of the sclera, 2) the evaluation of the interplay between input parameters and their effect on the deformation, as well as the strength of the correlations between input and output parameters and 3) a comparison of the results from the FE model with the results from experimental air-puff deformation imaging.

#### 2.2.1 Evaluation of individual input parameters: sensitivity study

The purpose of this study was to assess the type of relationship that exists between the input parameter and the output parameters (proportional, inversely proportional, or no relationship), as well as the magnitude of the change. The study was conducted according to the following procedure: five input parameters were assigned the initial values from Section 2.2.1. The remaining input parameter was set as a variable and evaluated in the FE model over the range described in Section 2.2.1. The stress-free configuration was used for all evaluations. This procedure was repeated so that each input parameter was evaluated as a variable. Output parameters were calculated for all solutions. The change in the output parameter over the evaluated range of the input parameter was calculated as a percent change.

#### 2.2.2 Simultaneous evaluation of all input parameters: random sampling study

The purpose of this study was to assess which input parameters have a stronger correlation to the output parameters. In this study, and unlike the sensitivity study, all input parameters are evaluated simultaneously. A code was written to 1) generate a random set of input parameters from the ranges presented in Table 1 and 2) evaluate the FE model using this set, and 3) calculate the output parameters from the FE solution. The stress-free configuration was used for all evaluations. A total of 400 random sets were evaluated. From the resulting data, the coefficient of determination (R^2^) between each input parameter and each output parameter was calculated.

#### 2.2.3 Comparison of experimental measurements and FE model

The purpose of this study was to compare the FE model to experimental results of scleral APDI. We predicted the corneal response to air puff as a function of IOP in the model, and compared it against experimental measurements of corneal deformation in which the IOP was varied experimentally.

##### 2.2.3.1 Experimental set-up and protocol

All images were acquired using a custom-developed SSOCT system described in previously published work (Curatolo et al., 2020; Bronte-Ciriza et al., 2021). The system uses a Mach-Zender interferometer configuration, a dual balanced photodetector (PDB480C-AC, Thorlabs, United States), and a MEMS-based vertical cavity surface emitting laser swept-source (SL132120, Thorlabs, United States), centered at 1,300 nm. The 3 mm-aperture low coil impedance galvanometric scanning mirrors (Saturn 1B, ScannerMAX, Pangolin, United States) enable ultrafast scanning, which is critical to capture deformation events that last only tens of milliseconds. The system has an axial rate of 200 kHz, an axial resolution of 16 μm, a depth of field of 5.15 mm, a large transverse field of view of 15 mm, and an ultra-fast transverse scanning pattern repetition frequency of 1 kHz. Scleral deformation was induced through a repurposed industry-standard, non-contact tonometer air-puff unit (Nidek Co., Japan) that was placed between the sample and the OCT objective lens so that it was coaxially aligned with the OCT scanning beam.

**FIGURE 3:**
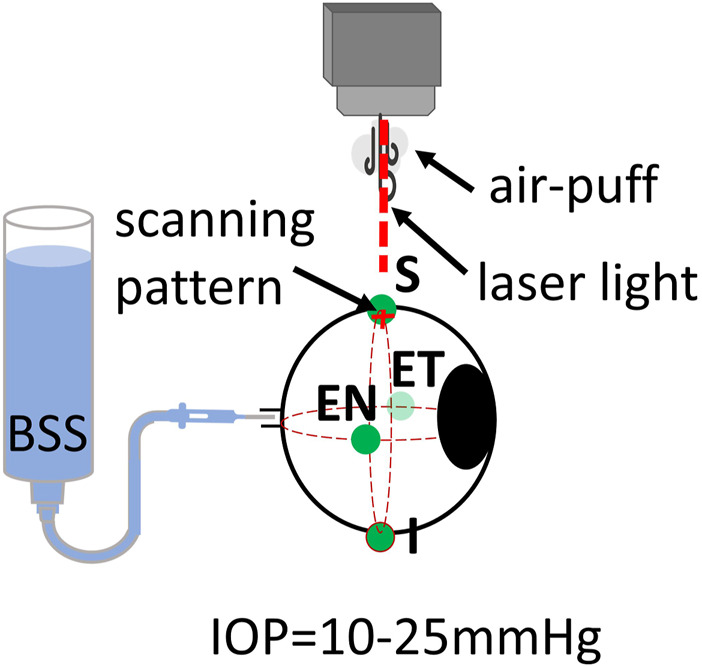
Schematic of inflation setup on rabbit eyes. Locations of air-puff excitation are Superior (S), Inferior (I), Equatorial Nasal (EN, and Equatorial Temporal (ET).

Three freshly enucleated rabbit eyes (adult New Zealand white rabbits) were obtained from a farm associated with the Veterinary School of the Universidad Complutense de Madrid (Spain), refrigerated at 4°C, and used within 48 h post-mortem. Muscles and conjunctival tissue were removed from the sclera of all eyes. Four different scleral locations were evaluated: superior (S), inferior (I), equatorial nasal (EN), and equatorial temporal (ET). A diagram of the positions can be seen in Figure 3.

All eyes were connected to an automated IOP control system with a needle through the optic nerve. The eye was then placed in a customized holder and aligned with the OCT laser scanning beam. Initially, the IOP was fixed at 10 mmHg. OCT measurements were collected over two orthogonal axes, each of which was 15 mm long. A complete measurement consisted of a total of 100 cross-axes scans, with each axis sampled by 64 A-scans. The total acquisition time of a complete data set was 100 ms (one cross-axes scan per ms). After a set of three measurements for each location, the eye position was carefully changed to the next location. All measurements were repeated for IOP of 15, 20, and 25 mmHg. Temperature and humidity conditions were kept constant throughout the measurements (at 21.0°C ± 2.0°C and 37.2% ± 1.1% respectively). The eye globe was kept moisturized with a balanced salt solution (BSS) with drops applied before and after each measurement.

##### 2.2.3.2 Data analysis

Data analysis included data processing and surface detection of the deforming ocular surfaces. The OCT images were obtained after standard image generation from wavenumber resampled spectra (Gora et al., 2009), using customized MATLAB routines. Air-puff excitation happens through a 2.4-mm-wide hole in a 5-mm-thick methacrylate window, which leads to an optical path difference at the center of the OCT images due to the difference in the refractive index. This difference was corrected using piece-wise registration routines written in ImageJ (National Institutes of Health, Bethesda, Maryland, United States). After obtaining OCT images, the ocular surfaces were segmented using a customized MATLAB routine (Bronte-Ciriza et al., 2021). For the analysis, only images from the sagittal meridian scanned were used.

##### 2.2.3.3 Finite element and inverse model

For the FE model, the apex displacement at an IOP of 10 mmHg was used as the starting point. For each of the positions in each eye, the material coefficient was estimated using an iterative process, such that the apex displacement at IOP = 10 mmHg in the simulation matches the corresponding experimental displacement. The FE model was then evaluated, for this material coefficient, at IOPs of 15, 20, and 25 mmHg. The simulated apex displacement at IOPs 15–25 mmHg was compared to the experimental values and the RMS error was calculated. The process was done for each position in each eye. In this procedure, the stress-free configuration is used only for IOP = 10 mmHg.

An inverse modelling procedure was programmed in MATLAB to estimate the material coefficient of each eye. First, a function f_apex_ was created. This function takes μ as input, and then calls ANSYS to solve the FE model. The FE model is solved such that the material coefficient is a variable, and the remaining input parameters are those described in Table 1, except for IOP, which is given a value of 10 mmHg.

The function calculates the apex displacement from the FE solver data. Then, the square of the difference between the simulated apex displacement and the experimental apex displacement at IOP = 10 mmHg is calculated. This number is the output of the function.

Then, the *fminbnd* MATLAB function was used to minimize the output of function X. *fminbnd* is a single-variable minimizer, bounded non-linear minimization based on golden-section search and parabolic interpolation. The boundaries were set to *μ* = 0.05–0.15. This minimization process yields a value of *μ* that was taken as an estimate of the material coefficient of the evaluated eye.

Using this value of μ, the FE model is evaluated for IOPs of 15, 20, and 25 mmHg, and the resulting apex displacement is calculated. The simulated results at 15–25 mmHg are compared to the experimental results at 15–25 mmHg for each eye.

## 3 Results

### 3.1 Sensitivity study

**FIGURE 4:**
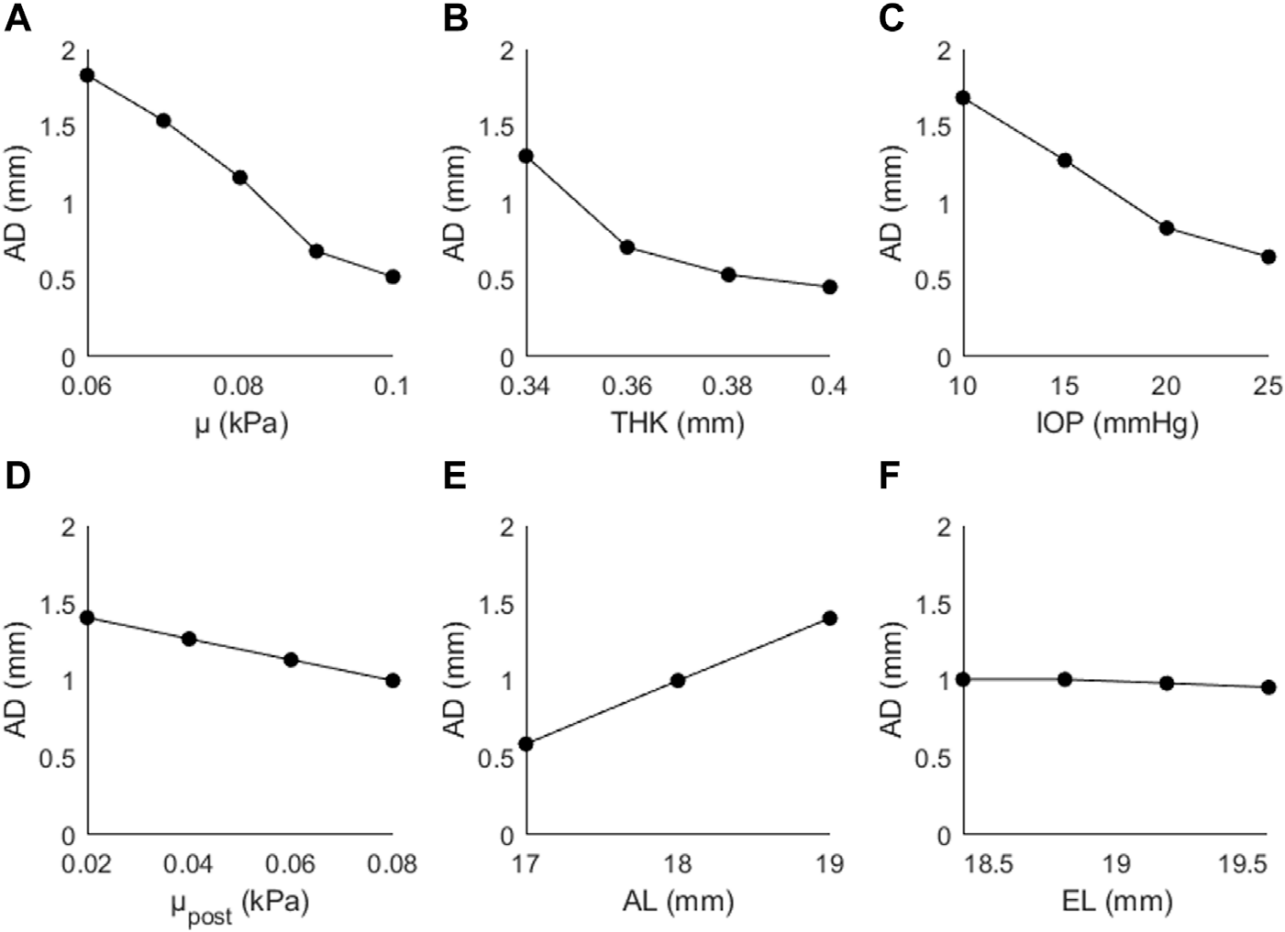
Apex displacement (AD) as a function of **(A)** the material coefficient µ, **(B)** thickness (THK), **(C)** intraocular pressure (IOP), **(D)** material coefficient µpost, **(E)** axial length (AL), and **(F)** the equatorial length (EL).

The results of the sensitivity study are presented for each output parameter (AD, CPR, AR). Figure 4 shows the predicted changes in AD as a function of μ, THK, IOP, μ_post_, AL, and EL. Values in parentheses indicate the percentage increase/decrease from the initial value of the input parameter to the highest investigated value. AD decreased as a function of μ (↓72%), THK (↓65%), IOP (↓61%), μ_post_ (↓29%), and EL (↓5%), and increased as a function of AL (↑138%). The observed changes were greater for the direct parameters (μ, THK, IOP) than for the two indirect parameters (μ_post_, EL). AL had the greatest effect on AD, and was the only parameter that showed a proportional and not an inversely proportional effect.

**FIGURE 5:**
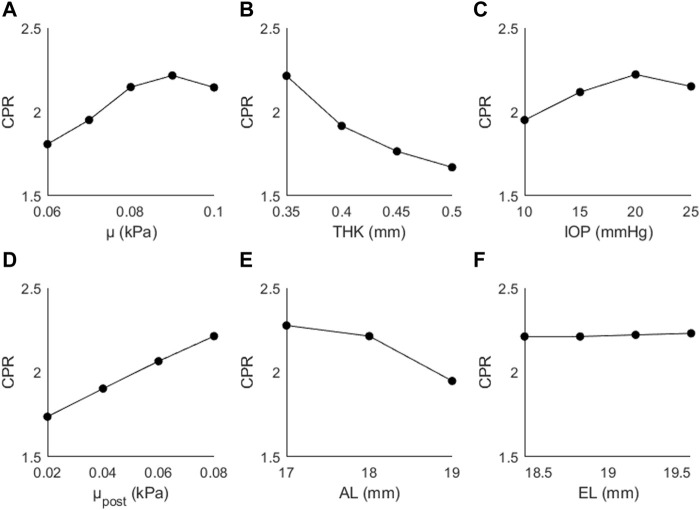
CPR as a function of **(A)** the material coefficient µ, **(B)** thickness (THK), **(C)** intraocular pressure (IOP), **(D)** material coefficient µpost, **(E)** axial length (AL), and **(F)** the equatorial length (EL).

Figure 5 shows the changes in CPR as a function of (A) μ, (B) THK, (C) IOP, (D) μ_post_, (E) AL, and (F) EL. The trends were not as consistent as those seen with AD; CPR increased as a function of μ (↑19%), IOP (↑10%), μ_post_ (↑27%), and EL (↑1%), and decreased as a function of THK (↓9%) and AL (↓14%). The largest changes are seen for the material coefficients, particularly the posterior coefficient.

**FIGURE 6:**
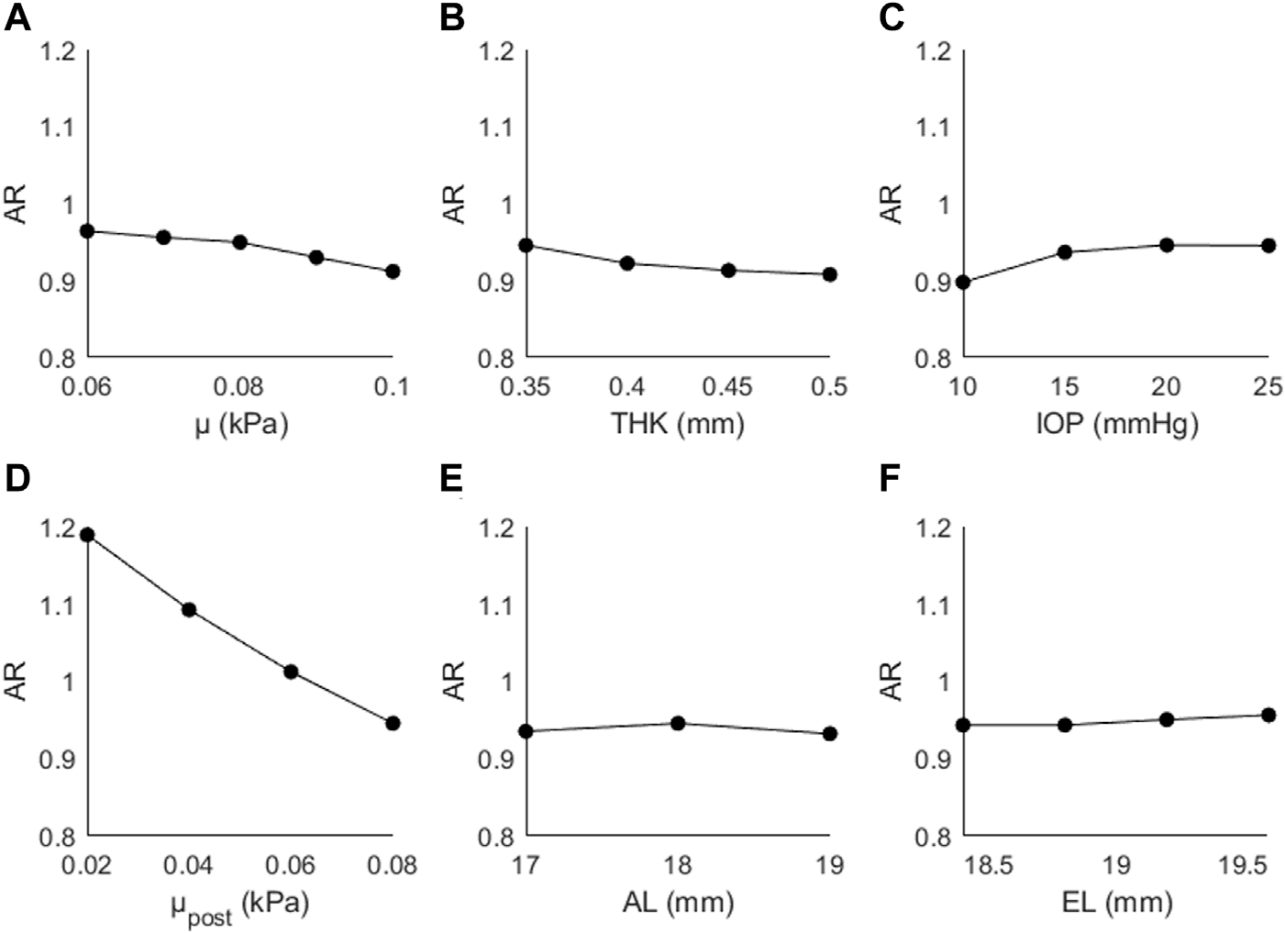
Changes in AR as a function of **(A)** the material coefficient µ, **(B)** thickness (THK), **(C)** intraocular pressure (IOP), **(D)** material coefficient µpost, **(E)** axial length (AL), and **(F)** the equatorial length (EL).

Figure 6 shows the changes in AR as a function of (A) μ, (B) THK, (C) IOP, (D) μ_post_, (E) AL, and (F) EL. AR decreased as a function of μ (↓5%), THK (↓2%), μ_post_ (↓20%), and AL (↓1%), and increased as a function of IOP (↑5%) and EL (↑1%). The generally small magnitude of the changes suggests that this parameter is primarily affected by the posterior material coefficient.

### 3.2 Random sampling study

**FIGURE 7:**
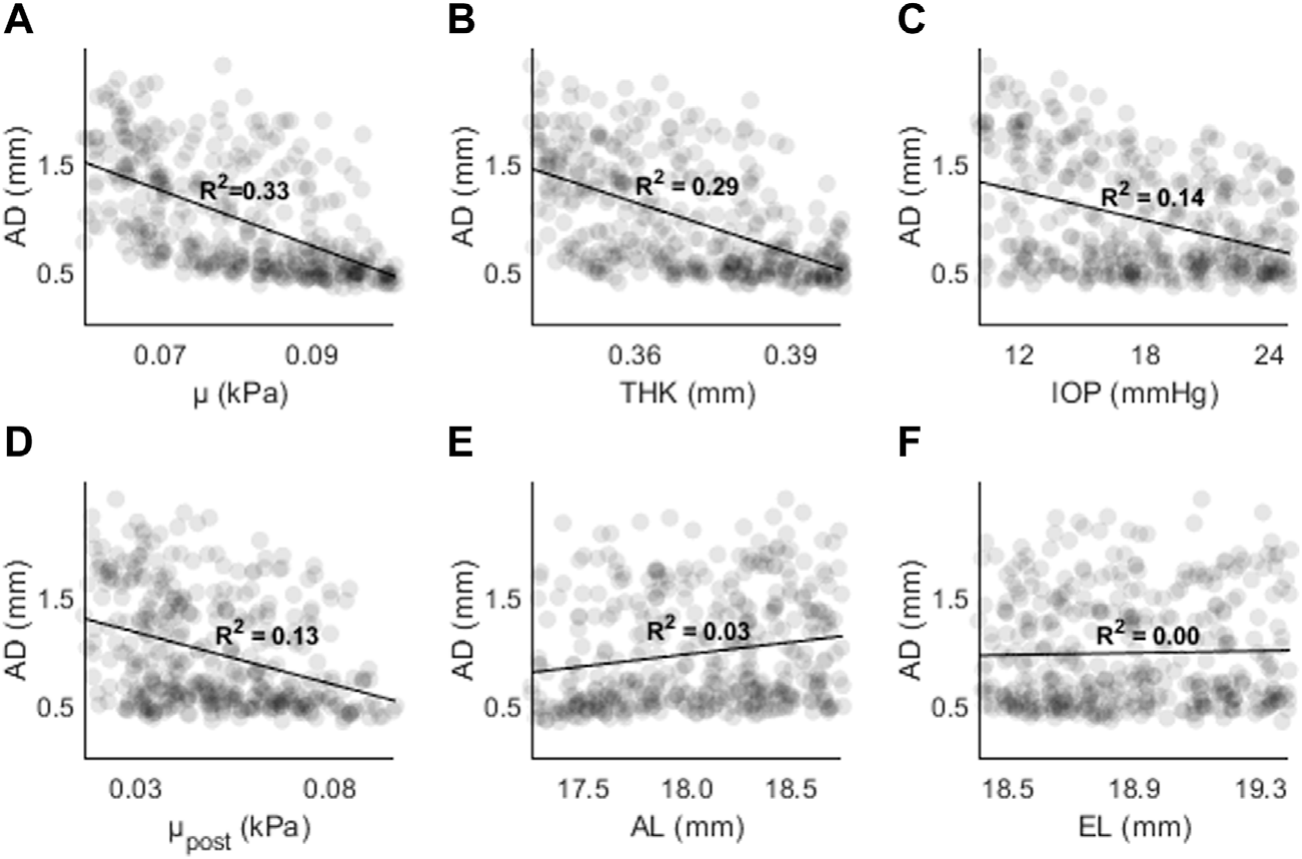
AD as a function of **(A)** the material coefficient µ, **(B)** thickness (THK), **(C)** intraocular pressure (IOP), **(D)** material coefficient µpost, **(E)** axial length (AL), and **(F)** the equatorial length (EL).

**FIGURE 8:**
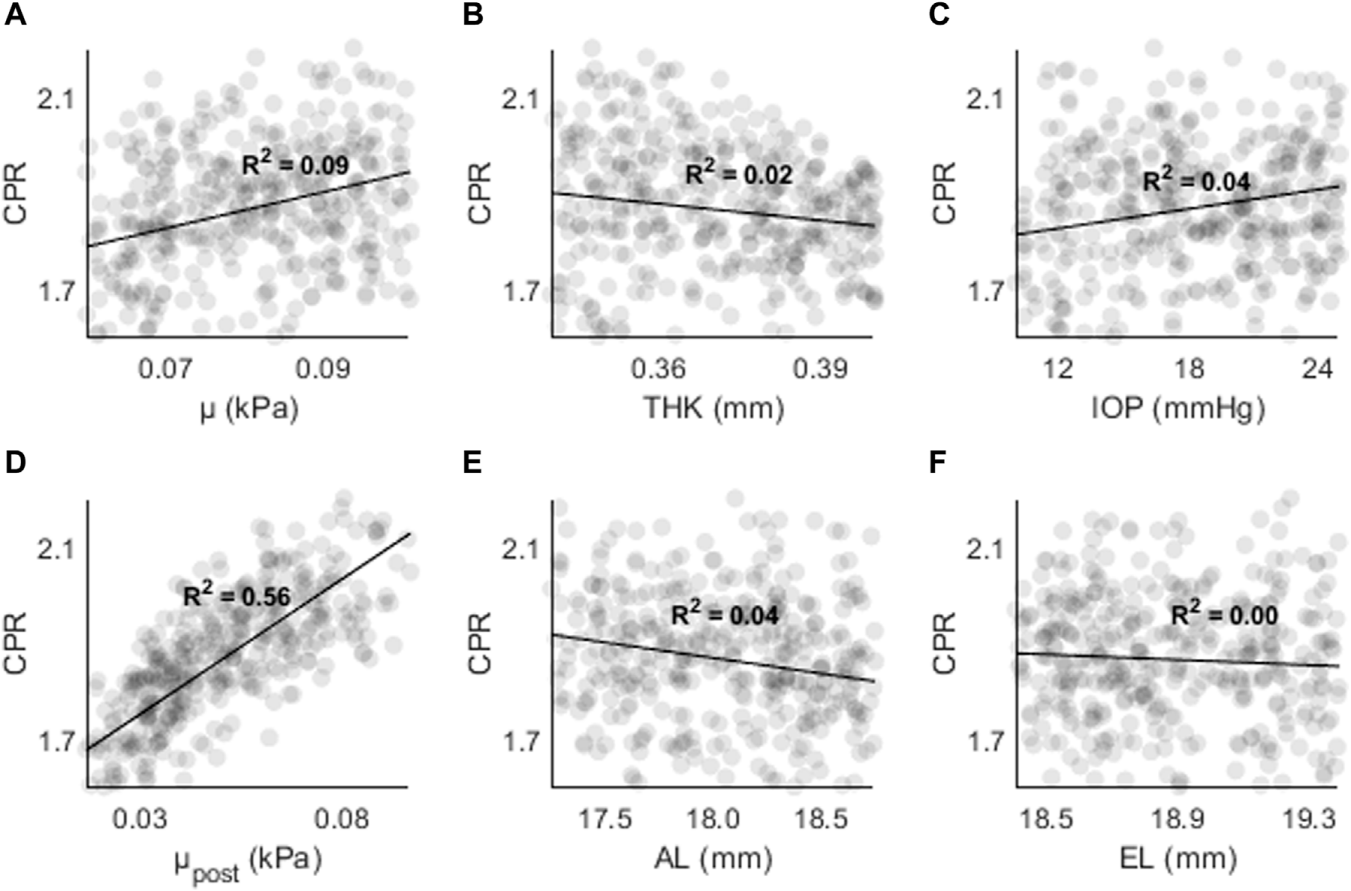
CPR as a function of **(A)** the material coefficient µ, **(B)** thickness (THK), **(C)** intraocular pressure (IOP), **(D)** material coefficient µpost, **(E)** axial length (AL), and **(F)** the equatorial length (EL).

**FIGURE 9:**
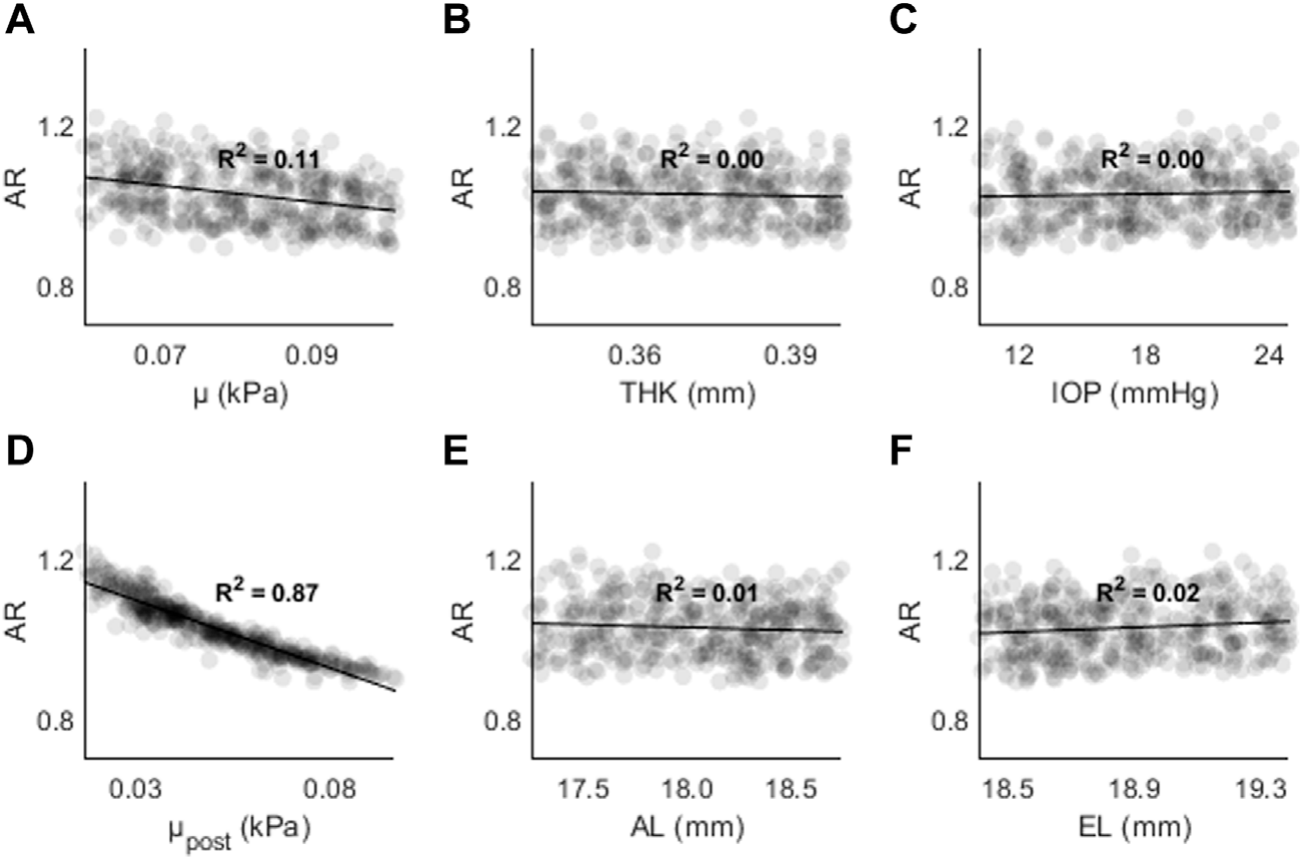
AR as a function of **(A)** the material coefficient µ, **(B)** thickness (THK), **(C)** intraocular pressure (IOP), **(D)** material coefficient µpost, **(E)** axial length (AL), and **(F)** the equatorial length (EL).

The results of the random sampling study are presented in Figures 7–9. Figure 7 shows AD as a function of (A) μ, (B) THK, (C) IOP, (D) μ_post_, (E) AL, and (F) EL. The strongest correlations were found for μ (R^2^ = 0.33, *p* < 0.01), followed by THK (R^2^ = 0.29, *p* < 0.01), IOP (R^2^ = 0.14, *p* < 0.01) and μ_post_ (R^2^ = 0.13, *p* < 0.01). The correlations for AL and EL were weaker (R^2^ < 0.05). Compared to the sensitivity study, the effects of IOP and AL were reduced, with the latter showing almost no correlation.

Figures 8, 9 shows CPR as a function of (A) μ, (B) THK, (C) IOP, (D) μ_post_, (E) AL, and (F) EL. R^2^ values are lower overall (R^2^ < 0.05), except for μ_post_ (R^2^ = 0.56, *p* < 0.01) and μ (R^2^ = 0.09, *p* < 0.01). The results are consistent with those found in the sensitivity study, where μ and μ_post_ had the greatest effect on CPR.

Figure 9 shows AR as a function of (A) μ, (B) THK, (C) IOP, (D) μ_post_, (E) AL, and (F) EL. The highest R^2^ values were found for μ_post_ (R^2^ = 0.87, *p* < 0.01) and μ (R^2^ = 0.11, *p* < 0.01). For all other input parameters, R^2^ < 0.05. The results are consistent with those found in the sensitivity study, where μ and μ_post_ resulted in the largest changes to AR.

The highest R^2^ values were found between μ_post_ and CPR/AR parameters. For AD the R^2^ values were stronger for μ and THK. Overall, the results show that parameters with higher spatial resolution (CPR, AR) are less likely to have dependence on non-material parameters. The results also show that other indirect parameters such as AL and EL are not strongly correlate with AD, CPR, or AR in the presence of other changes. THK and IOP are strongly correlated with AD, but not with CPR and AR.

### 3.3 Comparison between simulation and experimental data

**FIGURE 10:**
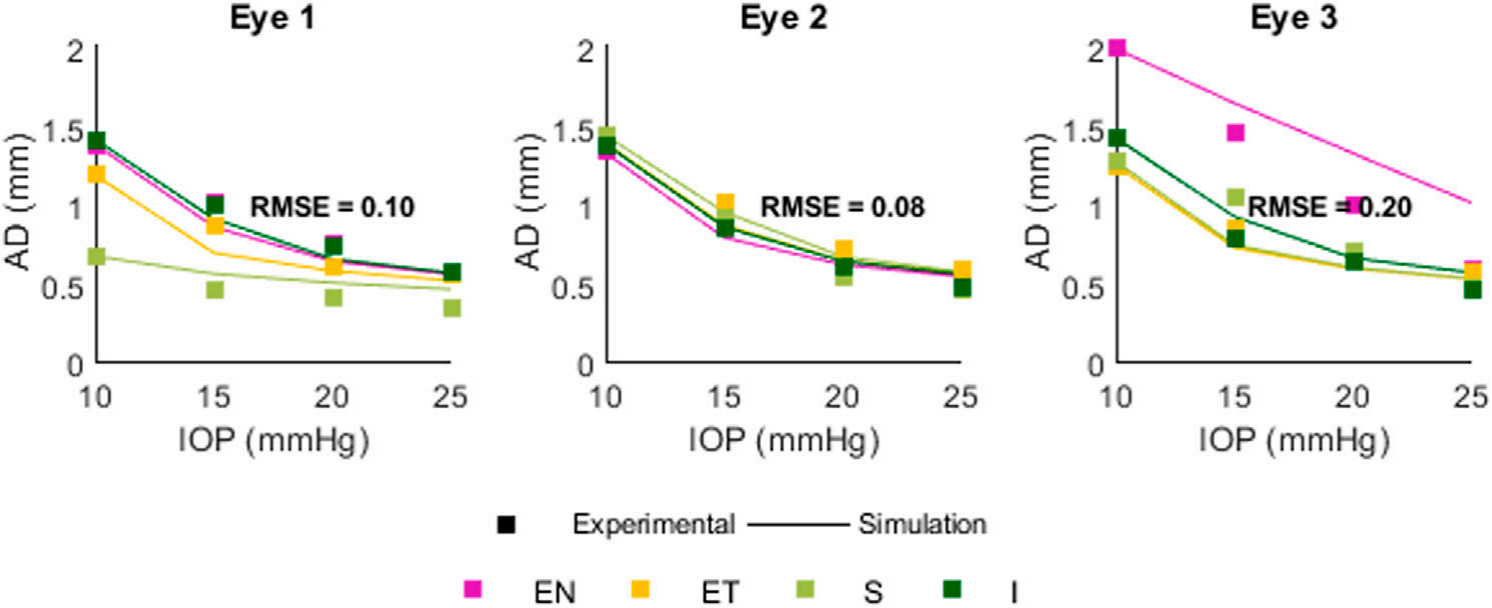
Comparison between experimental and simulated AD as a function of IOP. Positions are Superior (S) Inferior (I) Equatorial Nasal (EN) and Equatorial Temporal (ET). The RMSE for each eye was 0.10, 0.08, and 0.20 mm.

**FIGURE 11:**
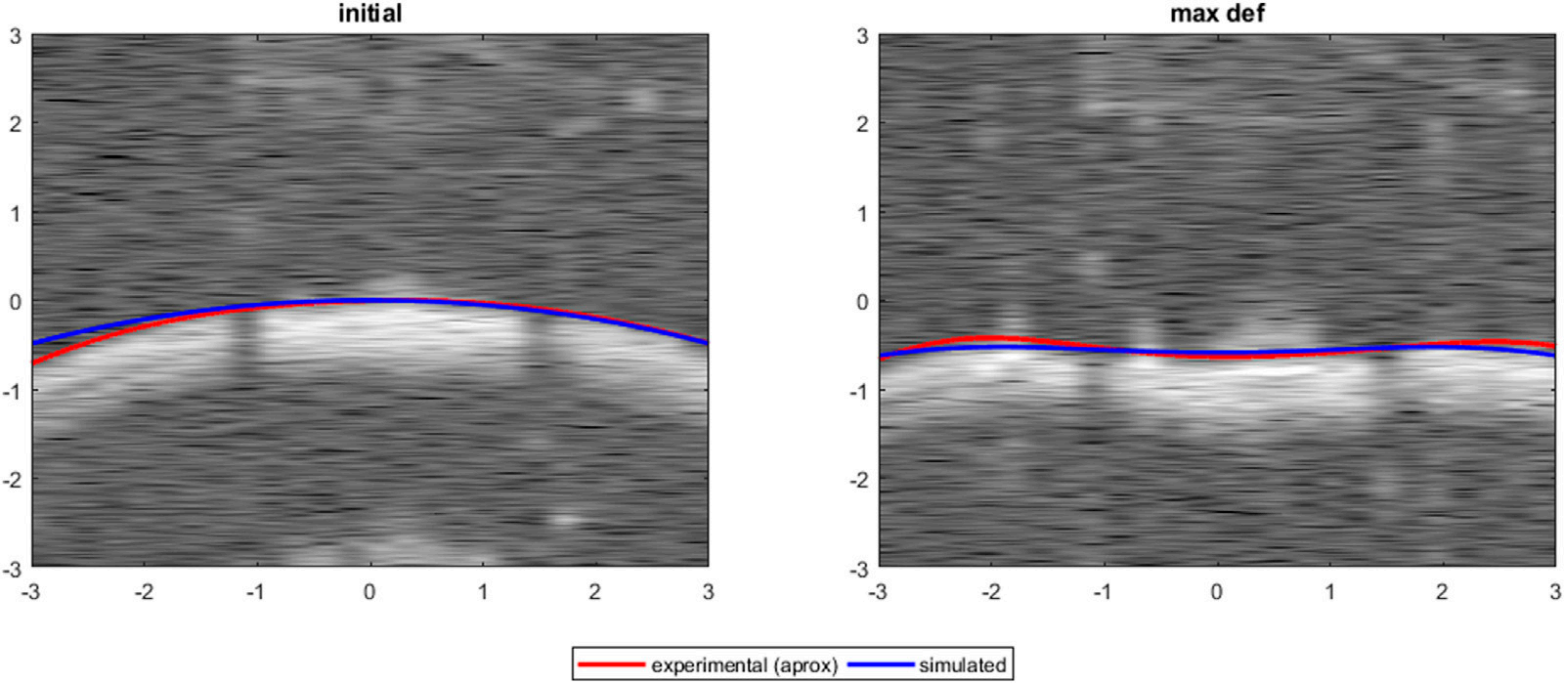
Experimental profile from OCT image and simulated profile for Eye 1, ET, IOP = 20 mmHg.

Figure 10 shows a comparison between experimental (squares) and simulated (lines) apex displacements as a function of IOP at different scleral locations. There was good correspondence between experimental and simulated apex displacements from IOP = 15 mmHg–25 mmHg. The average RMSE was 0.10 mm for Eye 1, 0.08 mm for Eye 2, and 0.20 mm for Eye 3. The RMSE corresponds well with the standard deviation of the experimental displacements at IOPs 15–25 mmHg (0.17 mm for Eye 1, 0.08 mm for Eye 2, 0.18 mm for Eye 3), suggesting that the differences in estimation between eyes are affected by the precision of the experimental measurements. Figure 11 shows the OCT image, segmentation profile, and simulated profile for ET in eye 1 at 20 mmHg.

## 4 Discussion

In this study, a model of scleral APDI was developed and the deformation was evaluated as a function of different input parameters. Output parameter AD, which was derived from a single point, showed a larger dependence on all input parameters (μ, μ_post_, THK, IOP, AL, EL), while output parameters CPR and AR, which are derived from multiple points, showed dependence on material parameters (μ, μ_post_) but were less dependent on THK, IOP, AL, and EL.

The material coefficient μ_post_ was found to greatly affect the values of the parameters CPR and AR. These two parameters are more complex than AD, as they compare the deformation in two regions, one central to the air-puff load and one peripheral. The effect of μ_post_, and the limited effect of the remaining input parameters, suggests that CPR and AR could be used to estimate material properties away from the region of air-puff application. This is of particular interest for *in vivo* applications, as reaching the posterior sclera with an air-puff is not feasible. Instead, the spatial profile of equatorial scleral deformation could be used to estimate the posterior sclera’s stiffness.

The scleral response was most influenced by, in descending order, μ, μ_post_, THK, and IOP. This was true for both random sampling and sensitivity studies. AL had an influence on the output parameters in the sensitivity study, but the influence was largely absent in the random sampling study, suggesting that the strength of the parameter is largely offset by changes in thickness, IOP, etc. EL on the other hand did not have much influence over the outputs in either study. These results suggest that an estimation of the material properties of the sclera would be aided by approximate knowledge of the thickness of the sclera at the location of air-puff application and the IOP of the eye. The thickness could be estimated from the image obtained in scleral APDI provided that this is a visible region, as is done in corneal APDI. The IOP could be estimated from tonometry measurements of the cornea, where work has been done on estimating IOP without the influence of material and geometric properties (Joda et al., 2016).

The comparison between experimental and simulated AD as a function of IOP showed similar behavior in the FE model and real eyes: a decrease in the apex displacement with increasing IOP, occurring in a non-linear fashion. The estimation varied in each eye, with RMS error of 0.10, 0.08, and 0.20 mm, which likely resulted from the experimental variability. From the FE model, we have also determined that AD as a function of IOP will also be affected by the initial AL, EL, and THK values, as well as by material coefficient α (set as a constant in this model). In this work only the effect of IOP was compared in experimental and simulated results, as this parameter can be controlled *ex vivo*. A full validation would entail a comparison between output parameters and multiple variable inputs (thickness, material properties, globe dimensions, IOP) in order to verify the expected correlations. For full comparison, the material properties would need to be either independently assessed with another method (extensiometry, OCE, etc.) or estimated by association, e.g., to age.

APDI has been primarily used in the cornea, but a comparison can still be made between this study and the literature. In a FE parametric study of corneal APDI, Kling (Kling et al., 2014) found that the material modulus of the cornea, IOP, scleral modulus, and thickness had significant influence over AD, with a decrease of 90% in scleral modulus decreasing AD by 0.20 mm. Nguyen (Nguyen et al., 2019) found the same effect of scleral modulus on corneal AD, with a lower modulus resulting in higher AD. This is consistent with our model, where AD decreased by 0.4 mm after a 90% decrease in μ_post_ in the sensitivity study. Similar effects were seen for other parameters, for instance Kling found a decrease of 0.4 mm in AD with a change in IOP from 15 to 25 mmHg in the porcine cornea; Nguyen found a decrease of 0.142 ± 0.02 mm from 10 to 20 mmHg in an FE model and a decrease of 0.50 ± 0.14 from 10 to 20 mmHg *in vivo* in the human cornea. In our sensitivity study we found a decrease of 0.64 mm in AD from 15 to 25 mmHg.

In any FE parametric study, modeling choices will affect the outcomes. Although we have quantified changes and correlations between deformation response (AD, CPR, AR) and parameters such as μ and IOP, the strength of the correlations will be dependent on the range of values evaluated for each input parameter.

The random sampling analysis showed a high correlation between μ_post_ and AR, which is worth exploring. First, our model accounts for changes in μ from the equator to the most posterior part of the sclera, but does not account for anterior variation. Changes in material properties in the anterior sclera could affect the peripheral displacement 1, and therefore affect AR. Second, in this model we have used a linear function (Eq. 2) to model the spatial variation of the sclera. Although the sclera’s material properties are known to vary along the axial length of the eye, this variation might not follow a linear pattern, i.e., (Elsheikh et al., 2010) find larger changes from anterior to equatorial than equatorial to posterior. In addition, the pattern of variation could be different in subjects with a pathology or a stiffening treatment in the sclera. The choice of a uniform, linear function will affect the deformation profile, output parameters, and strength of the correlations found in this paper. Third, AR is sensitive to the precision of the two displacements. In an FE model the displacement data is highly precise; in an experimental or *in vivo* application, the precision of the displacement will be lower, and the AR estimate noisier and less accurate.

In this work, an isotropic constitutive model was used. This model is not mechanistic, and the material coefficient does not necessarily reflect an intrinsic fiber or extracellular matrix modulus. Corneal APDI has been successful at predicting corneal stiffness through the use of isotropic models, despite the cornea’s anisotropy. FE studies of anisotropic scleral models suggest that the extracellular matrix, which is modelled as isotropic, has a larger influence in inflation than the fibers, which are directional (Grytz et al., 2014; Coudrillier et al., 2015). This suggests that the isotropic approach is suitable for scleral APDI. Anisotropic models, while more accurate, still require simplifying assumptions, for example holding material coefficients constant across the studied tissue (Coudrillier et al., 2015; Schwaner et al., 2020). They also typically require complex, multi-step inverse modeling schemes, making their implementation outside the laboratory challenging. Nonetheless, anisotropic considerations in FE modeling of APDI could provide additional information to characterize the sclera from a small number of displacements.

An area where investigating posterior scleral stiffness is relevant is myopia. There are numerous reports in the literature from *ex vivo* eyes (mostly animal myopia models) showing a decrease in posterior scleral stiffness from material (Phillips and McBrien, 1995; Phillips et al., 2000; McBrien et al., 2009) and thickness (Curtin and Teng, 1958). APDI could be a useful tool to further develop computational mechanical models of the sclera to study the effect of the sclera in healthy and myopic eyes. Also relevant to myopia is the ability to assess changes in scleral tissue stiffness after cross-linking treatment (CXL) (Dotan et al., 2014; Liu et al., 2016). CXL has been traditionally used in treating the cornea for conditions such as keratoconus, and the stiffening effects on the corneal tissue have been observed using extensiometry (Cherfan et al., 2003; Kling et al., 2012) and APDI estimations (Kling et al., 2014; Bekesi et al., 2017). Stiffness changes have been measured in extensiometry of cross-linked scleral tissue (Vinas-Pena et al., 2022), suggesting that scleral APDI could be a useful tool to assess scleral CXL.

## 5 Conclusion

This FE work found that the material properties of the sclera (local and general), thickness of the tissue, and the IOP were the most influential factors for a single-point deformation parameter (apex displacement), with globe dimensions less influential. For multi-point deformation parameters (asymmetry ratio or peripheral ratio), the local and general material properties were most influential, with thickness and IOP less coupled to the response. Scleral APDI could serve as a tool for diagnostics of scleral pathologies that impact its material properties, such as myopia, and treatments that act upon such properties, such as scleral cross-linking.

## Data Availability

The original contributions presented in the study are included in the article/Supplementary Material, further inquiries can be directed to the corresponding author.
